# STRATEGIES TO AGE WELL: BUILDING A CREATIVE AND IMPACTFUL CAREER IN BRAIN HEALTH

**DOI:** 10.1093/geroni/igae098.1023

**Published:** 2024-12-31

**Authors:** Vonetta Dotson

**Affiliations:** Georgia State University, Atlanta, Georgia, United States

## Abstract

Dr. Vonetta Dotson is a gero-neuropsychologist with a program of a researched focused on understanding positive (e.g., exercise) and negative (e.g., depression) modifiers of brain health in diverse older adults in order to identify ways to reduce health disparities in cognitive and affective disorders in late life, thereby enabling every individual to age well. The work is designed to be translational in nature, with implications for the daily lives of older adults and for how we diagnose, treat, and prevent cognitive and affective disorders. The National Institute on Aging (NIA) and the Gerontological Society of American (GSA) have been instrumental in her professional accomplishments throughout her training and career, ranging from early GSA conference experiences and participation in the Technical Assistance Workshop (former name of the Butler Williams program) during graduate school, to her current NIA funding through various mechanisms including R01, R21, and U19 grants. This presentation will summarize Dr. Dotson’s scientific research accomplishments, the role of NIA and GSA in her career, and her more recent translation of her research to community outreach, including her start-up company, CerebroFit, that puts into action the latest research on lifestyle interventions for healthy brain aging and her book Keep Your Wits about You: The Science of Brain Maintenance as You Age, which provides lay audiences with science-based facts and practical tools to help readers develop healthy lifestyles to optimize their brain health.

